# A Two-Way Communication Between Apical Periodontitis and Various Systemic Disorders: A Narrative Review

**DOI:** 10.7759/cureus.81482

**Published:** 2025-03-30

**Authors:** Ripal V Shah, Niraj Kinariwala, Sonali Patel, Shruti Bhut, Foram Patel, Gracy Gelani, Vidhi Parmar, Dhwani Bhatia

**Affiliations:** 1 Conservative Dentistry and Endodontics, Karnavati School of Dentistry, Gandhinagar, IND; 2 Dental Surgery, Karnavati School of Dentistry, Gandhinagar, IND

**Keywords:** apical periodontitis, association, cytokines, inflammation, metabolic disease, systemic health

## Abstract

One prevalent inflammatory disorder that affects the mouth is apical periodontitis. It starts with an infection in the tooth's pulp chamber. The periapical bone may eventually deteriorate as a result of this infection spreading there. Systemic immune responses are triggered when inflammatory cytokines generated in periapical lesions and pathogens and their metabolites in the periapical tissues enter the bloodstream. Numerous systemic disorders may emerge as a result of this procedure. Although endodontic infections can be influenced by systemic disorders, there is ample evidence that these infections can also result in bodily changes that impair general health. Therefore, rather than being a limited oral problem, apical periodontitis may be associated with systemic illnesses. Furthermore, individuals with chronic inflammation-related diseases may experience hyperinflammatory states, which could influence the progression or outcome of apical periodontitis. The underlying mechanisms and the relationship between apical periodontitis and systemic diseases are still unknown. Even though this topic has been explored previously, new information has just come to light. This review's objective is to evaluate the body of knowledge regarding the evolving relationships between endodontic therapy, apical periodontitis, and systemic health. Gaining a better understanding of this relationship will help medical professionals identify risk factors and enhance the recovery of apical periodontitis and systemic illnesses.

## Introduction and background

Caries, unsuccessful dental operations, or traumas are common causes of dental pulp infections. Gram-negative anaerobic bacteria predominate in these polymicrobial illnesses [1]. It is difficult to get rid of bacteria once they have entered and established themselves in the dentin [2]. Vessels that enter and exit through the periapical foramen or foramina deliver blood to the dental pulp [3]. Complete pulpal necrosis and an infected root canal (endodontic infection) are typically the results of the infection progressing when the pulpal vascular system is damaged. The tissue around the tooth's apex, referred to as the "periapical" zone, has an immunological and inflammatory reaction as a result of the bacterial pathogens that are continuously present in an infected root canal [4]. A periapical lesion is the word used to describe the inflammatory reaction brought on by an endodontic infection [5]. Alveolar bone resorption and degradation in the periapical area are hallmarks of a chronic inflammatory mouth disease called apical periodontitis (AP) [6].

According to a 2021 systematic review and meta-analysis, 52% of adults globally have at least one tooth that is impacted by AP [7]. To control the immune response, AP involves the local production of several proinflammatory cytokines [8]. Like deep periodontal pockets in periodontitis, the infected root canal continues to be a source of bacteria if appropriate endodontic therapy is not received [9]. The localized infection idea, which was put forth in the early to mid-1900s, suggested that periapical lesions might be the origin of dental infections in patients who also had serious systemic illnesses [10, 11]. This idea states that oral cavity microbes might lead to inflammation and illnesses in other body areas by possible mechanisms: (1) Transient bacteremia-induced metastatic infection; (2) Immune system-damaged metastatic inflammation; and (3) Microbial toxin-induced metastatic harm [12].

The phrase "metabolic syndrome" refers to a group of conditions that impact a single individual through a dysregulated immune response and increased systemic inflammation and include elevated blood glucose, abnormal cholesterol, high blood pressure, and excess abdominal fat [13]. The silent ailment known as periodontitis is prevalent in middle-aged and older adults [14]. Similarly, between the ages of 40 and 64, 63% of individuals with diabetes (ages 18 to 79) receive their diagnosis [15]. On the other hand, people with dental caries, the primary cause of endodontic disorders, are typically younger than those with type 2 diabetes or cardiovascular disease [16]. About 82.1% of American people between the ages of 20 and 34 have dental caries, according to the Centers for Disease Control and Prevention (CDC) [17].

Examining and discussing the body of research on the reciprocal association between endodontic infections and systemic disorders is one of the objectives of this study. To repair the periapical tissues, endodontic treatment aims to eliminate the infection by eliminating bacteria from the root canal system by cleaning and shaping [18]. The relationship between apical periodontitis (AP) and systemic diseases, such as autoimmune diseases, cardiovascular disease (CVD), unfavourable pregnancy outcomes (APO), metabolic disorders, and psychiatric disorders, is summarized in this study. A greater emphasis on comprehending the connection between systemic disorders and apical periodontitis (AP) would help doctors and dentists develop innovative treatment plans and promote the successful recovery of both conditions.

## Review

Effects of apical periodontitis on normal systemic health

There is limited proof in the studies that systemic disorders and apical periodontitis (AP) are connected. The goal of human and animal studies, however, is to identify systemic molecular changes in AP patients who do not have any other systemic diseases. Some research have focused on the effects of AP on resistance of insulin, which increases the possibility of diabetes [19]. For instance, Astolphi et al. found that AP may change insulin signalling and sensitivity in serum and skeletal muscle using a rat model of pulp-induced AP. This is most likely due to elevated plasma TNF-α levels [20]. Pereira et al. found that AP triggered inflammatory pathways in muscle tissue, enhanced macrophage infiltration, and elevated serum levels of heat shock protein [21].

Cintra et al. used a rat model of exposed pulp with apical periodontitis to investigate systemic inflammatory markers. They discovered that four AP foci led to higher serum levels and lower NO synthase levels, but that one AP focus had no systemic effect on the expression of proinflammatory cytokines [22]. Later it was concluded four AP foci were linked with increased cytokine levels and decreased serum levels [23]. Zhang et al. also concluded that AP raised the levels of cytokines in the blood serum of rats causing irreversible liver damage and reversible changes in the aortic arch, myocardium, and spleen [24]. The authors finally concluded that AP could have a negative impact on overall health [25].

Inchingolo et al. demonstrated the outcomes of AP on oxidative stress of humans by stating that an imbalance between pro-oxidants and antioxidants can disrupt redox signaling or result in molecular damage [26].

Effects of apical periodontitis on diabetes

Various studies have focused on the reciprocal relationship between diabetes and apical periodontitis. Metabolic disorders have shown adverse outcomes on the onset and severity of apical periodontitis (AP) [27]. As concluded by Kohsaka et al. (1996) diabetic rats presented with larger lesions because of increased inflammation, root resorption, and bone loss near the apex [28]. Subsequent research has supported these conclusions, demonstrating that diabetes makes AP patients more prone to inflammation and bone loss. A study that revealed diabetes may increase IL-17 production at the AP site provides more proof that diabetes affects the inflammatory response in periapical tissues [29].

The relationship between the prevalence of AP and diabetes in humans has been the subject of numerous studies. After discovering that 81.23% of diabetic patients had AP in at least one tooth, compared to 58% of control participants, Iwama et al. came to the conclusion that type 2 diabetes is strongly linked to a higher prevalence of AP [30]. Marotta et al. found that AP was common in the untreated teeth of diabetic patients, which may suggest that diabetes raises a person's likelihood of developing AP [31]. Smadi's study supported this, showing that chronic AP was more common in diabetics, particularly those with poor glycemic control [32]. While certain evaluations, like the one carried out by Tibúrcio-Machado et al. showed no evidence of a connection between diabetes and AP, the trend indicates diabetes and an increased quantity of periapical lesions [33]. Additionally, diabetic patients with chronic AP tend to recover more slowly and have greater lesions than normoglycemic patients (Rudranaik et al.) [34].

The healing results of traditional endodontic treatment in individuals with and without diabetes have been studied in a number of investigations. Fouad found that patients with diabetes had greater levels of periradicular disease and periodontal disease symptoms after having endodontic therapy, and that patients on insulin had a propensity to have more flare-ups [35]. Furthermore, a lower percentage of treatment success was linked to diabetes. Segura-Egea et al.'s systematic review revealed that diabetics have higher chances of AP in teeth treated with endodontics, thus demonstrating that diabetes is a significant preoperative factor in predicting the outcome of root canal procedures [15]. According to Arya et al., the diabetes group's periapical healing was significantly worse at the 12-month follow-up, even though periapical scores improved in both diabetic and non-diabetic patients after therapy [36].

Additionally, recent research has shown that whereas diabetes affects the pathophysiology of AP, AP can exacerbate the systemic consequences of diabetes. An association between insulin resistance and local dental inflammation was suggested by Schulze et al.'s report of a patient whose glucose levels jumped during an aggravation of a combined endo-perio lesion [37]. Increased triglycerides, HbA1c, IL-17 levels, inflammatory cells, and platelets are a few examples of these systemic consequences. Animal studies have shown that AP can also affect how body weight is distributed, affecting organs like the heart, brain, and gonads. Moreover, AP has been linked to increased oxidative stress in diabetic rats, which boosts uric acid levels, a symptom of chronic kidney disease. These findings show that AP management may help reduce the systemic oxidative conditions caused by diabetes [38].

Research on humans, including studies by Sánchez-Dominguez et al. [23] and Arya et al. [36], which discovered a link between higher HbA1c levels and worse periapical state in diabetic individuals, further supports the bidirectional association between endodontic infections and diabetes. Overall, these studies demonstrate the importance of endodontic therapy for the systemic health of diabetic patients.

Effects of apical periodontitis on cardiovascular diseases

Although few studies have examined it, researchers are interested in the possible systemic impact of apical periodontitis (AP) and its correlation with cardiovascular disease (CVD). These studies have examined disorders like hypertension, acute coronary syndrome, and coronary artery disease (CAD) [39].

Numerous research point to a connection between AP and heart conditions. Caplan et al. evaluated the association between coronary heart disease (CHD) and radiographically visible lesions of endodontic origin in 708 patients. Particularly in patients under 40, they found a link between AP and CHD [40]. Gomes et al. subsequently confirmed this, showing that midlife AP was a valid predictor of cardiovascular events [41]. However, among patients with unstable angina or acute myocardial infarction, AP and CHD did not correlate, according to a study by Pasqualini et al. [42]. On the other hand, Costa et al. discovered that chronic AP raised the incidence of CAD by 2.79 times in a cross-sectional analysis of 103 individuals having coronary angiography [43]. The risk of cardiovascular disease was 5.3 times higher for people with AP than for those without [44]. Additional research by Liljestrand et al. also found a connection between AP and cardiovascular disease; Liljestrand et al. explicitly noted a separate relationship between AP and CAD, namely acute coronary syndrome [45]. This emphasizes how crucial dental health is in determining total cardiovascular risk.

Regarding hypertension, Segura-Egea et al. discovered that tobacco usage can worsen the relationship between high blood pressure and the existence of AP [1]. Even though there is evidence linking AP to cardiovascular illnesses, more evidence is necessary to completely understand these connections. When Rashmi et al. examined inflammatory indicators in hypertension patients, such as fibrinogen, IL-6, and C-reactive protein (CRP), they found that AP changed the systemic levels of these markers [46].

In conclusion, additional study is necessary to clarify the precise mechanisms at work and identify potential therapeutic targets for both ailments, notwithstanding the evidence linking endodontic infections to cardiovascular problems.

Effects of apical periodontitis on atherosclerosis

The start of atherosclerosis is the main process in the development of the two most prevalent types of cardiovascular disease (CVD) [47]. Atherosclerotic plaques, which impact the inner, middle, and outer layers of arteries, such as coronary arteries, are the result of this inflammatory process [48]. Lipid buildup, connective tissue, and inflammatory, endothelial, and smooth muscle cells make up these plaques [48, 49]. The development, progression, and rupture of atherosclerotic plaque - which can result in thrombosis and major consequences like myocardial infarction and stroke - are all influenced by inflammation [50].

Atherosclerosis's primary cause is endothelial dysfunction which is a chronic inflammation, which can be brought on by pathogenic agents like microbes or cardiovascular risk factors like high LDL cholesterol, hypertension, hyperlipidemia, cigarette toxins, free radicals or a mix of these [51]. Because of this malfunction, the endothelial cells become more permeable, which permits LDL to move into the artery wall. Following their oxidation, these LDL particles release phospholipids that cause inflammation and draw monocytes to the area. These monocytes develop into macrophages, which take up the oxidized LDL and produce foam cells, which help to create fatty streaks [52]. Additionally, vascular soluble adhesion molecules are upregulated during this phase. This makes it easier for T-lymphocytes and monocytes to migrate into the arterial wall. The liver produces more C-reactive proteins (CRP) as a result of these cells' secretion of pro-inflammatory cytokines [53]. This causes an atheroma to develop over time, with a thin fibrous cap covering a necrotic core composed of various types of cells [54]. The fibrous cap weakens as the process progresses, weakening the plaque and possibly causing thrombus development, which can obstruct blood vessels and result in peripheral artery disease, myocardial infarction, or stroke [55].

Atherosclerosis may develop and worsen as a result of chronic apical periodontitis (CAP) in a number of ways [56]. First, bacteremia may allow endodontic bacteria to directly penetrate the artery walls, causing adaptive immunological responses and local inflammation that may result in the formation of atherosclerotic plaques. Also, endodontic infections release bacterial byproducts which lead to endothelial dysfunction and exacerbate the inflammatory process that causes atherosclerosis [57].

Effects of apical periodontitis on pregnancy

Apical periodontitis has been known to cause severe problems to pregnant women because of systemic inflammatory stress. Research indicates that hormones such as Prostaglandin E2 and TNF are released by inflammatory periodontal tissues. These substances enter the placenta and amniotic fluid and cause premature delivery [58]. Recent researches have also demonstrated the relationship between apical periodontitis (AP) and poor pregnancy outcomes. Apical periodontitis is associated with an increased risk of premature delivery, intrauterine growth restriction, and shorter pregnancy duration in postpartum women [59].

According to Khalighinejad et al. (2017), preeclampsia - a frequent pregnancy complication characterized by hypertension and proteinuria - which occurs after the 20th week of gestation may be strongly predicted by maternal apical periodontitis [60]. One of the main reasons for maternal death is preeclampsia [61]. The research that is now available about the relationship between maternal apical periodontitis and unfavourable pregnancy outcomes was checked in a recent systematic review conducted by Jakovljevic et al. (2021) [62]. These results confirm that early detection and management of apical periodontitis could lower the risk of preeclampsia and low birth weight preterm babies.

Effects of apical periodontitis on autoimmune disorders

In autoimmune diseases, inflammation occurs because of the immune system's reaction to the body's own tissues [63]. Examples are inflammatory bowel diseases (IBD), such as Crohn's disease and ulcerative colitis, psoriasis (Ps), and rheumatoid arthritis (RA) [64]. Apical periodontitis has been noted more commonly in those with autoimmune diseases like RA and IBD [65]. In a recent study conducted by Ideo et al in 2022, apical periodontitis was more common in those with autoimmune illnesses (RA, Ps, and IBD) than in healthy controls [66]. The cause of this can be the overproduction of various inflammatory cytokines which are connected to the onset and persistence of several disorders [67].

RA is linked to the onset of apical periodontitis, due to the advancement of RANKL-Osteoprotegerin pathway [68]. Medications that are used to treat autoimmune diseases include conventional Disease-Modifying Anti-Rheumatic Drugs (cDMARDs) and biologic Disease-Modifying Anti-Rheumatic Drugs (bDMARDs). bDMARDs target inflammatory pathways by inhibiting T and B cell receptors [69].

Piras et al. in 2017 concluded that apical periodontitis was more common in teeth of autoimmune disease patients treated with bDMARD [70]. In a similar study by Karataş et al. in 2020, AP was common in those taking biologic drugs [71]. But according to another research by Cotti et al. (2015, 2018), individuals taking biologic medications who had undergone endodontic treatment for apical periodontitis recovered faster than controls. This concluded that immune-modulating treatments may have an impact on how quickly apical periodontitis heals [72]. In conclusion, autoimmune diseases and immune-modifying drugs can affect the occurrence of apical periodontitis as well as the outcome of endodontic therapy.

Effects of apical periodontitis on psychiatric disorders

With estimates indicating that 22.5% of Americans may encounter mental health problems at some point in their lives, psychiatric disorders are on the rise [73]. The endocrine and immunological systems can be upset by stress, negative emotions, and sleep disturbances, making people more susceptible to long-term conditions such as apical periodontitis (AP) [74]. Mental health conditions also impair dental hygiene, which exacerbates conditions like AP and periodontitis [75].

For instance, severe depression may result in immune system disruption, increased adrenaline, and inflammatory marker activation, all of which reduce resistance to oral infections [76]. Patients with AP also frequently experience sleep issues and mental stress, which frequently prompts them to seek emergency dental care [77]. According to studies, mouth bacteria such as P. gingivalis and Fusobacterium nucleatum may even have an effect on brain function, causing neuroinflammation that connects oral disease to depression [78].

Because mental stress causes the release of stress hormones like cortisol, which impair immune function and raise inflammation, it is intimately linked to the severity of AP [79]. Adrenergic blockers can help lessen the effects of stress, which has been proven to exacerbate AP by encouraging bone resorption and cytokine release. The severity of AP is exacerbated by this imbalance between inflammatory chemicals and cortisol [80].

Although there is evidence linking AP to psychiatric problems through immunological and hormonal disturbance, further research is required to completely comprehend this association. Healthcare professionals should give patients with psychiatric problems the proper care and education while keeping their dental health in mind.

Effects of apical periodontitis on other systemic conditions

While a lot of research has been done on the association between diabetes and cardiovascular diseases and apical periodontitis (AP), other systemic diseases have also been studied for potential reciprocal links. Blood urea levels and AP are significantly correlated, with 73% of patients with last-stage renal disease (ESRD) having AP and 40% in the control group, according to Khalighinejad et al. [57]. Since there was no obvious cause-and-effect correlation, AP therapy might be considered in the treatment of ESRD.

Compared to the general population, women with inflammatory bowel disease (IBD) who were taking immunomodulators had larger lesions and a greater prevalence of AP (Piras et al.) [70]. According to Grønkjaer, patients with cirrhosis and AP had more cirrhosis-related issues and lower albumin levels and higher CRP [81]. Gomes-Filho et al. concluded that hypoestrogenia may worsen the development of AP and promote bone resorption by increasing osteoclast activity [82].

According to these investigations, there are several reciprocal connections between AP and systemic illnesses. Treating the source of illness may assist in improving general systemic health, but further research is required.

## Conclusions

A large number of research comprising epidemiological studies, clinical trials, and animal studies has checked the connection between apical periodontitis (AP) and systemic illnesses. To assess the patient's condition, develop a treatment plan, and diagnose AP dentists should consider medications and systemic illnesses. Physicians should be priorly aware of the periapical health of patients with systemic diseases to avoid the negative effects of AP. Routine follow-ups between physicians and dentists are essential for monitoring patient health and improving treatment outcomes for both AP and systemic illnesses.

This study concluded that bacterial inflammation along with apical periodontitis may negatively affect general health and contribute to conditions like cardiovascular diseases (CVDs), poor pregnancy outcomes, and metabolic dysregulation in diabetics. Although there is currently less information available on the same that those patients with diabetes or autoimmune illnesses may experience worse outcomes after endodontic therapy because of the incidence of apical periodontitis. Additionally, there is strong evidence that endodontic therapy might improve systemic health and reduce inflammation caused by apical periodontitis. More researches on the same are needed to show positive outcomes of endodontic therapy on systemic health in order to support these findings.
